# A multicentre prospective double blinded randomised controlled trial of intravenous iron (ferric Derisomaltose (FDI)) in Iron deficient but not anaemic patients with chronic kidney disease on functional status

**DOI:** 10.1186/s12882-021-02308-y

**Published:** 2021-03-30

**Authors:** S. Bhandari, V. Allgar, A. Lamplugh, I. Macdougall, P. A. Kalra

**Affiliations:** Hull University Teaching Hospitals NHS Trust and Hull York Medical School, Hull Royal Infirmary, Anlaby Road, Hull, HU3 2JZ UK

**Keywords:** Anaemia, Chronic kidney disease (CKD), Ferric derisomaltose, Iron deficiency, Randomised trial

## Abstract

**Background:**

Iron deficiency (ID) is common in patients with chronic kidney disease (CKD). Intravenous (IV) iron in heart failure leads to improvement in exercise capacity and improvement in quality-of-life measurements; however, data in patients with CKD are lacking.

**Methods:**

The Iron and the Heart Study was a prospective double blinded randomised study in non-anaemic CKD stages 3b-5 patients with ID which investigated whether 1000 mg of IV iron (ferric derisomaltose (FDI)) could improve exercise capacity in comparison to placebo measured at 1 and 3 months post infusion. Secondary objectives included effects on haematinic profiles and haemoglobin, safety analysis and quality of life questionnaires (QoL).

**Results:**

We randomly assigned 54 patients mean (SD) age for FDI (*n* = 26) 61.6 (10.1) years vs placebo (*n* = 28; 57.8 (12.9) years) and mean eGFR (33.2 (9.3) vs. 29.1 (9.6) ml/min/1.73m^2^) at baseline, respectively. Adjusting for baseline measurements, six-minute walk test (6MWT) showed no statistically significant difference between arms at 1 month (*p* = 0.736), or 3 months (*p* = 0.741). There were non-significant increases in 6MWT from baseline to 1 and 3 months in the FDI arm. Haemoglobin (Hb) at 1 and 3 months remained stable. There were statistically significant increases in ferritin (SF) and transferrin saturation (TSAT) at 1 and 3 months (*p* < 0.001). There was a modest numerical improvement in QoL parameters. There were no adverse events attributable to IV iron.

**Conclusion:**

This study demonstrated a short-term beneficial effect of FDI on exercise capacity, but it was not significant despite improvements in parameters of iron status, maintenance of Hb concentration, and numerical increases in functional capacity and quality of life scores. A larger study will be required to confirm if intravenous iron is beneficial in iron deficient non-anaemic non-dialysis CKD patients without heart failure to improve the 6MWT.

**Trial registration:**

European Clinical Trials Database (EudraCT) No: 2014-004133-16

REC no: 14/YH/1209

Date First Registered: 2015-02-17 and date of end of trail 2015-05-23

Sponsor ref R1766 and Protocol No: IHI 141

**Supplementary Information:**

The online version contains supplementary material available at 10.1186/s12882-021-02308-y.

## Significance statement

It is not known if administration of 1000 mg of intravenous iron versus placebo solution improves the six-minute walk test (6MWT) in patients with established chronic kidney disease (CKD) stages 3b-5, who are iron deficient but not anaemic.

This prospective double blinded randomised, multi-centre study in 54 patients who were given either a single dose of 1000 mg of intravenous iron or placebo solution showed that intravenous iron led to:

- a significant increased serum ferritin and transferrin saturation
- maintenance of the haemoglobin concentration
- an increased 6MWT from baseline to 1 and 3 months post infusion, but these changes were not statistically significant compared with placebo
- no adverse events attributable to intravenous iron.

This study shows that intravenous iron repletes iron but is insufficient to significantly improve the 6MWT. A larger study would be advisable in this patient group.

## Background

Patients with non-dialysis dependent chronic kidney disease (CKD) G3b or worse have a significant excess mortality, particularly from cardiac related causes, because of a spectrum of macrovascular structural changes, metabolic abnormalities in calcium and phosphate, hypertension, iron deficiency, anaemia and volume overload [1, 2], and a high proportion have or develop heart failure. In patients with heart failure and CKD, the prevalence of anaemia is as high as 32% [3] and survival was worse in patients with iron deficiency [3] suggesting iron may be an important substrate for optimal cardiac function and reduction in cardiac risk. Several other studies in heart failure patients [4–6] have also shown that IV iron may have beneficial effects on the heart which may impact on reduced cardiovascular events, improved functional capacity and improved symptom burden. Iron is important in a number of biological processes [7–11]. The potential underlying beneficial mechanism, which may in part relate to changes in skeletal energetics [12], at least in heart failure patients, has not been examined in a specific renal population. In addition, increased iron repletion appears to decrease the risk of mortality and cardiac events as seen in the PIVOTAL study in patients receiving haemodialysis [13, 14], and higher doses also seem more efficacious in patients with non-dialysis dependent CKD [15, 16].

We hypothesized that treatment with a single dose of 1000 mg of intravenous (IV) (ferric derisomaltose/iron isomaltoside 1000 (FDI); Monofer®) would benefit non-anaemic patients with CKD and iron deficiency in comparison to placebo over 1 and 3 months post infusion in terms of functional capacity.

## Methods

### Trial design and oversight

We conducted this prospective, randomised, double blinded, controlled trial at 3 sites in the United Kingdom [17]. The trial protocol was approved by the relevant health authorities and institutional review boards, and all the patients provided written informed consent. The trial sponsor (Hull University Teaching Hospitals NHS Trust) performed regular safety surveillance. Data were analyzed by the Department of Statistics, at the University of York, UK.

This was an academic investigator–led trial which was funded by Kidney Research UK and supported by an unrestricted grant from Pharmacosmos A/S (which also provided ferric derisomaltose/iron isomaltoside for the trial).

This study was carried out in accordance with Good Clinical Practice guidelines, the Declaration of Helsinki, and received ethical approval from the Northern Regional Ethics Service (NRES) Committee Yorkshire & The Humber - Leeds East, UK), approval reference number REC no: 14/YH/1209.

### Study participants

Adults with established non dialysis dependent CKD stages G3b-5 who had a serum ferritin (SF) of less than 100mcg/L and/or a transferrin saturation (TSAT) of less than 20% but no anaemia (defined in this study as a haemoglobin (Hb) 110-150 g/L for both males and females) were eligible to participate. The full eligibility criteria are provided in the protocol [17].

### Randomisation, treatment, and follow-up

The randomisation was performed by a computer program (sealedenvelope.com). Labels were consecutively numbered 1–60. These labels were then sealed in non-transparent double sealed envelopes. Access to these envelopes was not available to blinded investigators. Details of the iron therapy were held by pharmacy - the interventional iron therapy was matched with the relevant randomisation number.

We randomly assigned participants, in a 1:1 ratio, to receive a 100 ml infusion of either 1000 mg of IV iron (FDI) or 100 ml of placebo solution in a blinded fashion as described in the methods paper and below [17]; patients were evaluated at baseline, 1 and 3 months. Managing clinicians treated patients’ CKD according to standard practice.

In brief the placebo was 100 ml of 0.9% saline. This was a clear solution, and the iron solution was brown/black. For the intervention (experimental) arm, FDI 1000 mg was dissolved in 100 mL of normal saline 0.9% and given over 30 min as a single IV infusion. For the comparator (placebo) arm, 100 mL of normal saline 0.9% was given over 30 min as a single IV infusion. Participants were closely monitored throughout the infusion and for an additional 30 min after infusion to detect haemodynamic changes or other adverse effects.

Blinding was based on the double screen technique (the patient sits with their arm through the split in the double screens). The screen was also covered by a bed sheet to ensure the research nurse/Doctor and patient cannot see through the split. This was requested by the ethics committee such that the patient could not see the infusion, but the person giving the infusion could so that they could ensure no adverse skin reactions as the arm was not covered. This person administering the infusion was not part of the study analysis as they were not blinded. This technique ensured the double-blind nature while maintaining safety for the patient.

### Study procedures

These are detailed in the methods paper [17] but in brief patients underwent assessments at baseline, 1 month and 3 months. These consisted of the 6-min walk test (6MWT), laboratory measurements including eGFR and biochemical profile (BCP), full blood count (FBC), haemoglobin (Hb), serum ferritin (SF), transferrin saturation (TSAT), C-reactive protein (CRP) and NT-ProBNP (Roche diagnostics) and cystatin C. Quantification of proteinuria was carried out by measurement of urinary protein:creatinine ratio (uPCR) or if diabetic, urinary albumin:creatinine ratio (uACR) depending on availability and use in the recruiting centres. New York Association Functional classification (NYHA), electrocardiogram (ECG) and 2D echo were carried out in all Group 1 patients at baseline, 1 and 3 months. Two questionnaires: the Kidney Disease Quality of Life Short Form Questionnaire (KDQoL-SF-36) and The Minnesota Living with Heart Failure Questionnaire (MLHF) were completed at the three time points for Groups 1. Pulse wave velocity (PWV) and augmentation index (AiX) measurements were performed at baseline and at months 1 and 3 for Groups 1.

### Trial endpoints

The primary endpoint was based on functional capacity at 1 and 3 months, assessed by the 6MWT, in patients receiving a single dose of 1000 mg of IV FDI or infusion of placebo. This required 48 participants (24 per group) to detect a 25 m (m) increase in 6MWT between the intervention and control group with 80% power at a 5%, 2-sided significance level. Although the 6MWT has not been validated in a CKD population, it was selected as the primary endpoint in the Peritoneal Dialysis Heart Failure Study funded by the British Heart Foundation [18], and perhaps more importantly, the Health Technology Assessment (HTA) funded BICARB study (bicarbonate in elderly patients with CKD) which have both finished. In addition, it was used as an endpoint in the FAIR-HF study (in heart failure patients) [19], which was used for calculation of sample size as detailed in the methods paper [17]. In brief calculations from the FAIR-HF study, using a Mann-Whitney U test and assuming a mean 6MWT in the control group of 274 m and equal SD in each group of 30 m [5], suggested that a study of 90 participants (45 per group) would permit the detection of a 25 m increase in 6MWT between the intervention and control group with 90% power at a 1%, 2-sided significance level (Stata version 11.0). This estimate was powered at 90% rather than 80%, and this group has used a significance of 1%, rather than the more conventional 5%. Therefore, using this estimated sample size calculation taken from a heart failure group, the calculated sample size for the current proposed study assuming a mean 6MWT in the control group of 274 m and equal SD in each group of 30 m, a study of 48 participants (24 per group) would permit the detection of a 25 m increase in 6MWT between the intervention and control group with 80% power at a 5%, 2-sided significance level (NQuery version 6). Allowing for trial attrition of approximately 10% we aimed to recruit 54 participants to yield a final evaluable sample of 48 participants (2per group) for the primary study.

Secondary endpoints consisted of efficacy endpoints which included changes in SF, TSAT and Hb concentrations, and 2 questionnaires: KD-QOL SF-36, and MLHF.

Safety endpoints included death, infections, vascular access thrombosis, hospitalization for any cause, and hospitalisation for infection, each assessed during the whole study period. Data on serious adverse events were collected prospectively, and events were coded with the use of the *Medical Dictionary for Regulatory Activities* (MedDRA), version 15.1. Data on non-serious adverse events, other than infection and vascular access thrombosis, were not collected.

### Statistical analysis

All data was analysed in accordance with an intention-to-treat principle wherein data from any randomised participant was included in the arm to which they were randomised. Continuous laboratory parametres were summarized using mean (standard deviations), or median with interquartile ranges (25th and 75th percentile). Categorical variables were summarized using number (%). For the primary outcome measure, 6MWT, ANCOVA was used to compare groups at 1 month and 3 months, adjusting for baseline 6MWT. The absolute change from baseline to the 1 month and 3 is presented as mean (SD) mANCOVA was also used for the secondary outcomes. A *p*-value of < 0.05 was considered to indicate statistical significance. All analyses were undertaken using IBM SPSS Statistics for Windows, version 26 (IBM Corp., Armonk, N.Y., USA).

## Results

### Patients: baseline characteristics

The patient disposition consort figure was published previously [17] but in brief, of the 316 patients who were screened for entry into the trial, 261 did not meet the criteria for randomisation, declined to participate or were not eligible. One patient consented but did not enter the study. A total of 54 patients were randomly assigned to the main study group (26 patients to FDI and 28 patients to the placebo) and constituted the intention-to-treat population. Follow-up was complete for all patients except 2 who missed their final follow-up visit, but all patients did not carry out all tests.

The characteristics of the 2 arms of the study group were similar at baseline, although there was a 4 years age difference with the FDI group (*n* = 26; mean (SD) age 61.6 (10.1) years) vs the placebo group (*n* = 28; mean (SD) age 57.8 (12.9) years). Mean (SD) serum creatinine was 167.0 (40.2) vs. 204.9 (67.3) micromol/L and eGFR 33.2 (9.3) vs. 29.1 (9.6) ml/min/1.73m^2^ at baseline in FDI and placebo treated patients, respectively. There was a difference in the mean (SD) baseline distance covered during the 6MWT (386.6 (135.8) metres in FDI vs 414.7 (104.5) metres in placebo treated patients; Table 1).

**Table 1:** Baseline demographic data; Mean (Standard deviation (SD)), number (n) or percentage (%) for the total for all of group 1 and unblinded data for the two randomised arms; uACR/uPCR = urinary albumin/protein:creatinine ratio. eGFR = Estimated glomerular filtration rate; CRP = C-Reactive Protein. 6MWT = Six-minute walk test. MLHF = Minnesota Living and Heart Failure Questionnaire. Electrocardiogram (ECG): N = Within normal limits; NCS = Abnormal and not clinically significant; ACS = Abnormal and clinically significant. FDI = Ferric Derisomaltose

Group and number of participants	FDI or Placebo ***n*** = 54 CKD, no anaemia but iron deficiency	FDI ***n*** = 26	Placebo ***N*** = 28
**Age** (years) (SD)	59.6 (11.7)	61.6 (10.1)	57.8 (12.9)
Range	32–78	37–78	32–78
**Sex**			
Male	26 (49%)	11 (42.3%)	15 (53.6%)
Female	27 (51%)	14 (53.8%)	13 (46.4%)
*Unknown*	1	1	0
**Ethnicity**			
White	42 (78%)	19 (73.1%)	23 (82.1%)
Asian	3 (5%)	1 (3.8%)	2 (7.1%)
Black	7 (13%)	4 (15.4%)	3 (10.7%)
Mixed Race	1 (2%)	1 (3.8%)	0 (0%)
Unknown/other	1 (2%)	1 (3.8%)	0 (0%)
**Smoker**			
Yes; current	5 (9%)	1 (3.8%)	4 (14.3%)
Yes; previous	17 (32%)	8 (30.8%)	9 (32.1%)
No	32 (59%)	17 (65.4%)	15 (53.6%)
**Body Mass Index** (BMI)	30.3 (6.5), 53	30.7 (6.8)	30.01 (6.4)
**Serum Ferritin** (SF) (microg/L)	66.3 (44.1) 53	64.2 (29.1) 26	68.4 (55.3) 27
**Transferrin Saturation %** (TSAT)	20.1 (7.4) 53	22.3 (8.8) 26	19.7 (5.6) 27
**Haemoglobin** (Hb) (g/L)	128.7 (10.1), 54	131 (7.4), 26	126.6 (11.8), 28
**Serum Creatinine** (micromol/L)	186.7 (58.6), 54	167 (40.2), 26	204.9 (67.3), 28
**eGFR** (ml/min/1.73m^2^)	31.1 (9.6), 54	33.2 (9.3), 26	29.1 (9.6), 28
**Cystatin C**	2.2 (0.6), 52	2.1 (0.5), 26	2.4 (0.6), 26
**uACR** (mg/mmol)	60.9 (133.3), 26	26.9 (40), 13	94.8 (181.4), 13
**uPCR (mg/mmol)**	83.8 (128.4), 40	51.9 (59.3), 19	112.7 (164.8), 21
**CRP** (mg/L)	5.0 (4.4), 53	6.3 (5.5), 26	3.8 (2.4), 27
**Serum Albumin** (g/L)	39.8 (5.6)	40.8 (4.2)	38.8 (6.9)
**Platelet count** **(X10**^**5**^**)**	235 (58.9)	226 (52.4)	243 (64.1)
**Phosphate** **(mmol/L)**	1.2 (0.2)	1.1 (0.2)	1.2 (0.2)
**NT pro BNP** **(ng/L)**	485.2 (1268.4), 51	422.5 (881.9), 25	545.4 (1569.5), 26
**PWV measurement**	8.3 (3.2), 54	8.3 (2.8), 26	8.3 (3.6), 28
**AiX measurement**	24.2 (10.7) 54	25.4 (10.7) 26	24.1 (10.8) 28
**6MWT** Metres (m)	401.2 (120.2), 52	386.6 (135.8), 25	414.7 (104.5), 27
**KD-KQoL SF36** **(normalised data)**	40.2 (10.4), 48	40.2 (10.4), 21	40.4 (9.55), 27
**MLHF**	23.97 (26.3)	22.7 (25.7) 18	25.0 (27.3) 24
**Systolic Blood Pressure** **(mmHG)**	133.6 (19.5), 54	138.2 (19.9), 26	129.4 (18.4), 28
**Diastolic Blood Pressure** **(mmHG)**	77.3 (11.3), 54	78.5 (10.7), 26	76.2 (11.8), 28
**Baseline ECG**			
**N**	38	17	21
**NCS**	13	8	5
**ACL**	1	1	0
**Not Recorded**	2	0	2
**Diabetes**	17	8	9
**Hypertension**	4	2	2
**Polycystic Kidney Disease**	5	2	3
**Pyelonephritis/Reflux**	3	1	2
**Glomerulonephritis / IgA/vasculitis/interstitial nephritis**	14	8	6
**Other/unknown**	11	5	6

### Primary endpoint: 6MWT

**Fig. 1:**
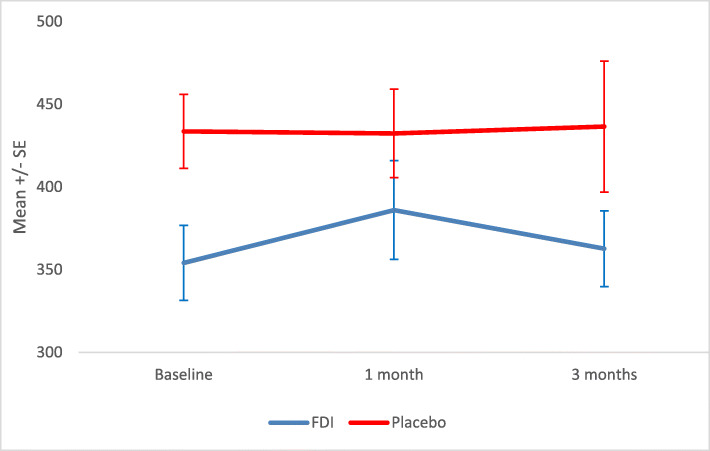
Mean and standard error (SE) 6-min walk test (6-MWT) at baseline, 1 and 3 months in the Ferric Derisomaltose (FDI) and placebo arms

Adjusting for baseline 6MWT, the 6MWT in the main study participants at 1 month and 3 months showed no statistically significant difference between FDI and placebo arms (*p* = 0.736 and 0.741 respectively). (Fig. 1). Analysis of change in 6MWT showed that the mean (SD) change for FDI patients from baseline to 3 months was 6.0 (89.1) metres compared to 1.9 (111.2) metres for the placebo arm patients (Suppl Table 1). During follow up there was a greater than 25 m increase in 6MWT observed in 8/22 (36.4%) of patients at 1 month and 10/20 (50%) at 3 months in the FDI arm vs 8/25 (32% - 1 month) and 10/24 (41.7% - 3 months) patients in the placebo arm, respectively (*p* = 0.753, *p* = 0.580).

### Secondary efficacy outcomes

**Fig. 2:**
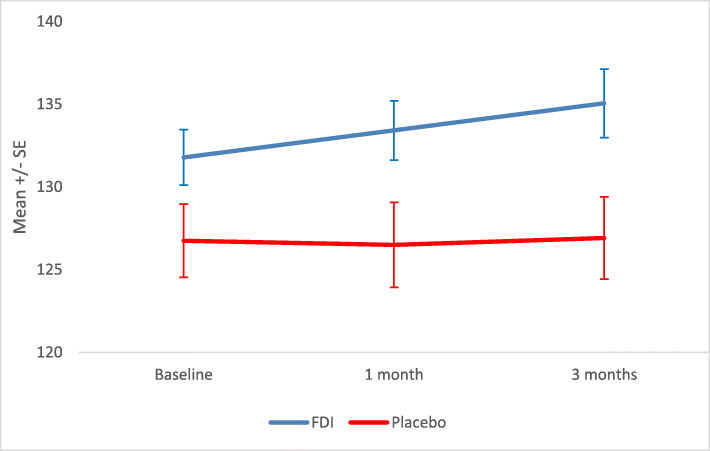
Mean and standard error (SE) of Haemoglobin (Hb) for IV iron (Ferric Derisomaltose) versus IV placebo at baseline, 1 month and 3 months

There was no statistically significant difference in Hb at 1 month (*p* = 0.195) and 3 months (*p* = 0.152) (Fig. 2 and Table 2). There was an upwards trend in Hb with an absolute mean increase of 1.7 g/L and 3.7 g/L with FDI compared to a fall of 1.0 g/L and 0.3 g/L in the placebo arm, thus, there was a difference of 2.7 g/L and 4.0 g/L in Hb in favor of FDI at 1 and 3 months, respectively.

**Table 2:** Summary of mean and standard deviation (SD) for haemoglobin (Hb) and haematinics; serum ferritin and transferrin saturation (TSAT) at baseline, 1 and 3 months

		FDI			Placebo			
		Mean	SD	n	Mean	SD	n	*P* value
Hb	Baseline	131.0	7.4	26	126.5	11.8	28	
	1 month	132.7	7.2	24	125.5	12.8	26	0.195
	3 months	134.7	8.9	20	126.2	12.4	25	0.152
Serum Ferritin	Baseline	64.2	29.1	26	68.4	55.3	27	
	1 month	266.0	105.8	23	70.5	55.8	26	< 0.001
	3 months	234.4	105.3	21	69.4	59.8	24	< 0.001
TSAT	Baseline	22.3	8.8	26	19.7	5.6	27	
	1 month	29.6	9.5	22	19.1	7.1	26	< 0.001
	3 months	26.4	10.5	21	18.0	6.8	23	< 0.001

**Fig. 3:**
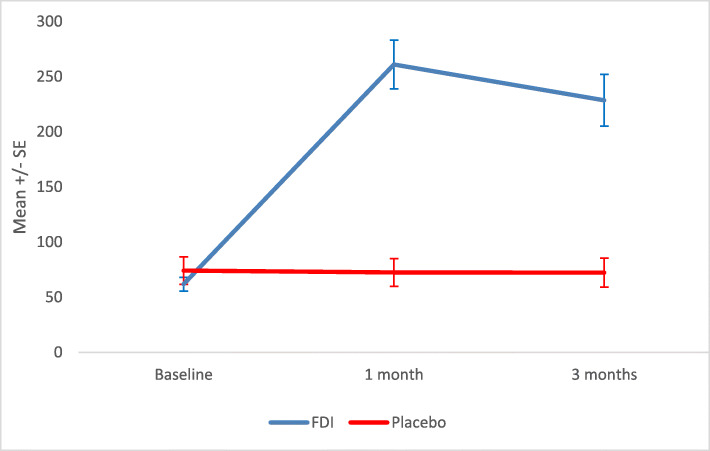
Mean and standard error (SE) of Haematinics – serum ferritin for IV iron (Ferric Derisomaltose) versus IV placebo at baseline, 1 month and 3 months

**Fig. 4:**
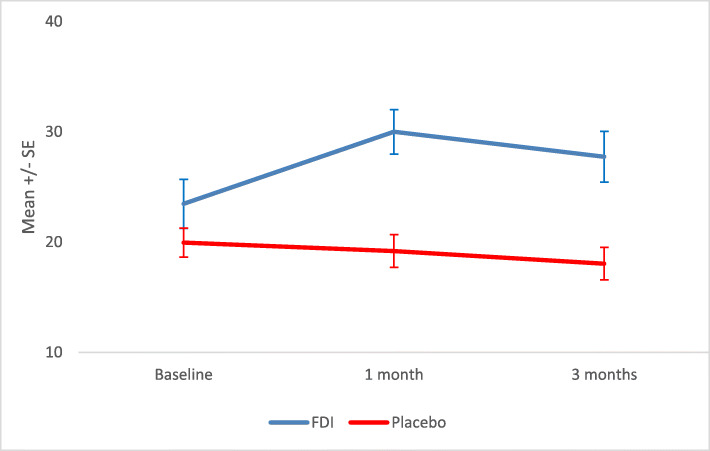
Mean and standard error (SE) of Haematinics –transferrin saturation (TSAT) for IV iron (Ferric Derisomaltose) versus IV placebo at baseline, 1 month and 3 months

There was a statistically significant difference for SF and TSAT at both 1-month (*p* < 0.001, *p* < 0.001) and 3 (*p* < 0.001 and *p* = 0.001) months, respectively, with greater increases in the FDI group (Figs. 3 and 4**,** Table 2)

Overall, 96 and 95% of participants achieved a SF >100microgram/L at 1 and 3 months in the FDI group in comparison to 19 and 21% in the placebo group, respectively (*p* < 0.001, *p* < 0.001). 77 and 67% of participants achieved a TSAT > 20% at 1 and 3 months in the FDI group in comparison to 23 and 30% in the placebo group, respectively (*p* < 0.001, *p* = 0.016).

#### Questionnaires

The MLHF questionnaire showed that those receiving IV iron had similar baseline scores to placebo in Group 1, and IV iron did not impact this score significantly (Table 1 and Suppl Table 2).

**Fig. 5:**
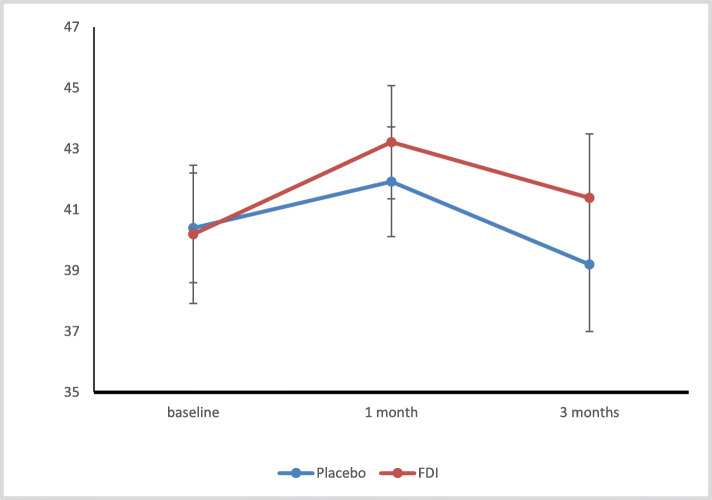
Mean and standard error (SE) for Kidney Disease Quality of life – Short Form-36 (KD-Qol SF-36) questionnaire (values are Z transformed and normalised values for component summary measure of Physical and Mental Health) for IV iron (Ferric Derisomaltose) versus IV placebo at baseline, 1 month and 3 months

The numerical improvements at 1 and 3 months in the normalized KD-QoL-SF-36 questionnaires in FDI patients were modest but not statistically significant. At 1-month, despite the numerical increases in normalised scores (difference of 1.5), there was no significant difference between the intervention arms (*p* = 0.981) (Fig. 5 and Suppl Table 3a). At 3 months, there was a larger difference (2.3) which was not significant between the two study arms (*p* = 0.451) (Fig. 5 and Suppl Table 3a). Repeated measures analysis across the three time points included 14 FDI and 23 placebo patients with complete baseline, 1-month and 3-month data and there was no statistically significant difference between the two arms (*p* = 0.515) (Suppl Table 3b).

### Safety analysis

#### Renal function

Renal Function as assessed by serum creatinine, eGFR and cystatin C were similar in both study arms with no statistically significant difference between FDI and placebo at 1 or 3 months. Proteinuria did not change with time or with IV iron treatment (Suppl Table 4).

#### Phosphate, albumin, platelets and CRP

Serum phosphate levels and platelet count remained stable with no significant changes throughout the study period in both FDI and placebo arms. Serum albumin was similar in both placebo and intervention study arms and did not change throughout the study. Mean (SD) CRP was unchanged throughout the study period; it was 4.2 (2.7) vs 6.9 (18.3) mg/L at 1-month (*p* = 0.6), and 7.5 (6.8) vs 10.3 (23.0) mg/L at 3 months (*p* = 0.46) in the FDI and placebo patients, respectively.

#### Blood pressure and endothelial function

Blood pressure remained stable over follow-up and was similar in both arms of Group 1 (Suppl Table 5). Endothelial function measured clinically using PWV and AiX did not change at 1 or 3 months post FDI infusion or in the placebo arm (Suppl Table 5).

### Adverse events

A total of 19 adverse events were documented in 13 participants during the duration of the study (Table 3). This equated to 19 events in 162 patient months in the whole cohort of Group 1. There were no treatment-related serious adverse events adjudicated by the primary investigators at each site, and no deaths, strokes or hospitalisations during the study period. There were also no hypersensitivity reactions or infusion reactions with FDI.

**Table 3:** Summary of adverse events

Adverse Events	Total	FDI	Placebo
Infections	5 Infections consisting of one urinary tract infection (UTI); 1 lower respiratory tract infection (LRTI), 1 flu and one exacerbation of chronic obstructive pulmonary disease (eCOPD) and one shingles flare	1 flu 1 UTI, 1 infective 1 eCOPD	1 LRTI 1 shingles
Cardiovascular events	Angina × 2	0	2
Shortness of breath	2	0	2
Other	Hypoglycaemia Had intravenous fluid for elective CT scan ? lung Cancer Gout Depression Per Rectal bleeding Acute Kidney Injury episode Cough Poor Blood Pressure control	0 0 0 1 1 0 0 1 1 2	1 0 1 0 0 1 1 0 0 0
	19 in 13 patients	9 in 6 patients	10 in 7 patients

### Cardiac assessments

Patients were classified into NYHA categories I to IV, and the average was similar in both placebo and FDI arms of group1. In total 3/18 (16%) and 3/22 (14%) patients had an improvement in NYHA category in the FDI arm compared to the placebo arm, respectively after 3 months. There was only one ECG which was deemed clinically significantly abnormal at baseline during the study, and this remained unchanged throughout the study in the placebo arm. No patient manifested a change in the status of the ECG. The standard 2-D echocardiogram results were also similar over time in the 2 randomised arms of the study. One echocardiogram was reported as abnormal and clinically significant at baseline in one patient and a further one in a second patient during the 3-month study period in the placebo arm of the study.

The mean (SD) NT-Pro BNP levels appeared to fall post FDI infusion (422.5 (881.9) pg/ml at baseline to 242.5 (209.1) pg/ml at 1 month) in comparison to placebo (545.4 (1569.5) pg/ml at baseline to 608.8 (1891) pg/ml at 1 month), but this did not reach significance (*p* = 0.371) (Suppl Table 6).

## Discussion

The Iron and the Heart Study investigated whether a single dose of 1000 mg of IV iron (ferric derisomaltose/iron isomaltoside 1000 (FDI); Monofer®) could improve exercise capacity in comparison to placebo over 1 and 3 months in non-anaemic CKD patients who had iron deficiency. Our results demonstrate several important findings in addition to the relationship between IV iron and functional capacity using the 6MWT, including quality of life.

The optimal management of iron deficiency in patients with non-dialysis dependent CKD is unclear with some evidence suggesting benefit of treatment [18]. In addition, evidence of functional improvement in non-anaemic iron deficient patients with CKD is lacking, as are head-to-head randomised controlled studies comparing IV iron versus placebo. In contrast to results from studies in heart failure patients [4–6], results of this trial showed no significant impact of IV FDI on 6MWT at 1 or 3 months post infusion, and only a modest but non-significant improvement in summary component score in QOL SF36 scores when compared with placebo. Haemoglobin remained stable and there were significant increases in SF and TSAT without significant changes in endothelial dysfunction, assessed by PWV.

The Kidney disease improving global outcomes (KDIGO) clinical practice guidelines recommends the use of iron therapy in CKD patients with a SF of <500micrograms/L or a TSAT ≤30% if it is desired to increase Hb or to reduce erythropoiesis stimulating agent therapy [19]. The National Institute of Clinical Health and Care Excellence (NICE) and the UK Renal Association have similar recommendations [20, 21]. A recent higher threshold of SF of 800micrograms/L, only for hemodialysis patients, based on data from the PIVOTAL trial has been included in the revised Renal Association guidelines, but this does not apply to this study for which we cannot make any recommendations except the need for a further study with sufficient power to see if higher thresholds might have an impact on functional status in non-dialysis CKD. Our results in this population indicate that the addition of 1000 mg of iron in patients with CKD and iron deficiency but no anaemia did not lead to a significant difference in 6MWT scores or quality of life scores.

These findings may be due to several factors including the sample size, dropouts and incomplete data collection, the relatively well-preserved cardiac function, and the relatively short follow-up period. In addition, the relatively well preserved 6MWT at baseline in retrospect might have indicated the margin for improvement may have been small in comparison to heart failure trials where the baseline values were more than 100 m less. Imputation using last observation carried forward was not significant. The dose of iron administered for iron repletion may have been insufficient to improve cellular energetics. Furthermore, there was an imbalance in baseline characteristics (age was greater and baseline 6MWT was lower in FDI patients compared with patients in the placebo group) and so we are cautious to infer any definitive conclusions.

Earlier work in heart failure patients has shown skeletal muscle improvements with IV iron [12], which may possibly not occur in uraemic CKD patients as a result of the presence of various uraemic toxins. The balance of effects of oxidative stress generated in chronic disease and with IV iron may have an impact although we did not see any additional impact of IV iron on clinical measures of endothelial dysfunction. Animal models suggest that in experimental CKD, in which iron deficiency may also be present, the impact of increased pro-oxidative factors is negated by antioxidant factors [22, 23]. Although the heart failure trials testing iv iron dosing included patients with renal impairment, the average eGFR in our population was 30 ml/min/1.73m^2^ and thus consistent with more advanced CKD. In animal models of experimental uraemia we have observed that there are marked changes in energetics, insulin resistance and mitochondrial function because of uraemia [10, 11, 24–26]. These may not have been reversible with a single dose of 1000 mg IV iron in the current study. The levels of NT Pro BNP fell post IV iron infusion in comparison to placebo, but the difference between the two groups did not reach statistical significance. Nevertheless, this may be an interesting signal as in a previous study of 40 patients with heart failure and modest renal impairment there was a significant reduction in NT Pro BNP with IV iron treatment [27]. These findings are in line with the trends observed in the current study.

Patients assigned to treatment with FDI were no more likely to experience an adverse event than those receiving placebo treatment. This trial was not designed to determine the mechanism of any therapeutic effect of IV iron but to determine whether there was a clinical functional benefit associated with its administration. However, this trial provides valuable information for the design of a future randomised controlled trial of sufficient power to answer the question definitively.

The strengths of our trial include its double-blind nature which reduced bias, and the multiple explorative analyses carried out to direct future research. Based on the data it demonstrates that a larger scale trial would be required to reliably confirm or refute a benefit of IV iron. Limitations of the trial include the modest size of the study and short follow-up. Thus, the generalisability of the trial findings is limited, and a larger study is needed. In addition, because quality-of-life data were missing for some patients, the interpretation of the effect of the iron dose with regard to these endpoints is limited. The trial was not designed or powered to detect effects on hard clinical endpoints, but the safety demonstrated in this study would add support to the conduct of an appropriately powered event-driven study examining the effects of IV iron on patient-related outcome measures.

## Conclusions

In summary, this randomised controlled trial showed that, among non-anaemic patients with CKD and iron deficiency, a single dose of 1000 mg of FDI had no statistically significant impact on functional capacity or endothelial function, a trend to improved quality of life and no adverse effects of treatment.

Given the increasingly recognised importance and clinical implications of iron deficiency, this study adds further information. It should be recognised that the benefit of IV iron may be dose-related and 1000 mg may be insufficient to produce the necessary effects that are seen in patients with heart failure. This has been noted in the recent NIMO study [28].

At present the literature concerning functional benefits of IV iron in patients with iron deficiency and non-dialysis dependent CKD disease is limited, based upon small open label studies. Although the intervention in the present study did not lead to a significant increase in 6MWT, perhaps because the groups were not ideally matched at baseline and the overall trial population was small, there was a trend and numerical improvement that could be further assessed in a future larger trial.

## Supplementary Information

**Additional file 1: Supplementary Table 1.** Summary of change in six-minute walk test (^MWT) baseline to 1 month and 3 months. **Supplementary Table 2a.** Summary of Minnesota Living with Heart Failure (MHLF) Questionnaire at baseline to 1 month and 3 months. **Supplementary Table 2b.** Summary of Restless legs score (RLS) Questionnaire at baseline to 1 month and 3 months. **Supplementary Table 3a and b.** Descriptive summary of Kidney Disease Quality of Life – Short form − 13 (KDQoL-SF) mean z transformed and normalised scores Questionnaire at baseline to 1 month and 3 months for total component summary measure (Physical Health and Mental Health individually and the total overall score). Initial analysis using ANCOVA and subsequent repeated measures analysis. **Supplementary Table 4.** Summary of Renal Function as assessed by serum creatinine (micromole/L). eGFR (ml/min/1.73m^2^), Cystatin C (g/L) and urinary proteinuria (mg/mmol) were similar in both groups and there was no significant change from baseline to 1 or 3 months. uACR – urinary abbumin;creatinine ratio; uPCR = urinary protein creatinine ratio. **Supplementary Table 5.** Summary of mean blood pressure (BP) in mmHG and Pulse wave velocity (PWV) measures including augmentation index (AiX) at baseline, 1 month and 3 months. **Supplementary Table 6.** Summary of Cardiac Biomarker; N terminal pro Brain natriuretic peptide (NT Pro BNP).

## Data Availability

All data that is anonymized will be available from Hull University Teaching Hospitals NHS Trust with the relevant permissions and agreement of the Research and Development Committee of Hull University Teaching Hospitals NHS Trust. The complete dataset is currently been analysed for post hoc studies.

## Abbreviations

AiX: Augmentation index
ANCOVA: Analysis of covariance
ANOVA: Analysis of variance
BCP: Biochemical profile
BMI: Body mass index
CKD: Chronic kidney disease
CRP: C reactive protein
ECG: Electrocardiogram
eCOPD: Exacerbation of chronic obstructive pulmonary disease
eGFR: Estimated glomerular filtration rate
FBc: Full blood count
FDI: Ferric derisomaltose
Hb: Haemoglobin
HTA: Health Technology Assessment
IV: Intravenous
ID: Iron deficiency
KDIGO: Kidney disease improving global outcomes
KD-QoL: Kidney disease quality of life
LRTI: Lower respiratory tract infection
MedDRA: Medical Dictionary for Regulatory Activities
MLHF: Minnesota Living with heart failure
NHS: National Health Service
NICE: National Institute of Clinical Health and Care Excellence
NRES: Northern Regional Ethics Service
NT-pro BNP: N terminal pro brain natriuretic peptide
NYHA: New York Heart Association functional classification
PWV: Pulse wave velocity
QoL: Quality of life
6MWT: Six-minute walk test
SD: Standard deviation
SF: Serum ferritin
TSAT: Transferrin saturation
uACR: Urinary albumin:creatinine ratio
UK: United Kingdom
uPCR: Urinary protein:creatinine ratio
USA: United States of America
UTI: Urinary tract infection

## References

[1] Go AS, Chertow GM, Fan D, McCulloch CE, Hsu CY (2004). Chronic kidney disease and the risks of death, cardiovascular events, and hospitalization. N Engl J Med.

[2] Rostand SG, Kirk KA, Rutsky EA (1984). Dialysis-associated ischemic heart disease: insights from coronary angiography. Kidney Int.

[3] de Silva R, Rigby AS, Witte KK, Nikitin NP, Tin L, Goode K, Bhandari S, Clark AL, Cleland JG (2006). Anaemia, renal dysfunction and their interaction in patients with chronic heart failure. Am J Cardiol.

[4] Okonko DO, Grzeslo A, Witkowski T, Mandal AK, Slater RM, Roughton M, Foldes G, Thum T, Majda J, Banasiak W, Missouris CG, Poole-Wilson PA, Anker SD, Ponikowski P (2008). Effect of intravenous iron sucrose on exercise tolerance in anaemic and nonanaemic patients with symptomatic chronic heart failure and iron deficiency FERRIC-HF: a randomised, controlled, observer-blinded trial. J Am Coll Cardiol.

[5] Anker SD, Comin Colet J, Filippatos G, Willenheimer R, Dickstein K, Drexler H, Lüscher TF, Bart B, Banasiak W, Niegowska J, Kirwan BA, Mori C, von Eisenhart RB, Pocock SJ, Poole-Wilson PA, Ponikowski P (2009). FAIR-HF trial investigators. Ferric carboxymaltose in patients with heart failure and iron deficiency. N Engl J Med.

[6] Ponikowski P, von Veldhuisen DJ, Comin-Calet J (2015). Beneficial effects of long term intravenous iron therapy with ferric carboxymaltose in patients with symptomatic heart failure and iron deficiency (CONFIRM-HF). Eur Heart J.

[7] Fairbanks V, Beutler E, Beutler E (2001). in Williams hematology.

[8] Dunn LL, Rahmanto YS, Richardson DR (2007). Iron uptake and metabolism in the new millennium. Trends Cell Biol.

[9] Bhandari S (2011). Risk factors and pathophysiology of metabolic change in uraemic cardiac disease. Front Biosci (Landmark Ed).

[10] Semple D, Smith K, Bhandari S, Seymour AM (2011). Uraemic cardiomyopathy and insulin resistance: a critical role for Akt. J Am Soc Nephrol.

[11] Taylor D, Bhandari S, Seymour AM (2015). Mitochondrial dysfunction in uraemic cardiomyopathy. Am J Physiol Ren Physiol.

[12] Charles-Edwards G, Amaral N, Sleight A, Ayis S, Catibog N, McDonagh T (2019). Effects of Iron isomaltoside on skeletal muscle energetics in patients with chronic heart failure and iron deficiency (FERRIC-HF II). Circulation..

[13] Macdougall IC, White C, Anker SD, Bhandari S, Farrington K, Kalra PA, McMurray JJ, Murray H, Steenkamp R, Tomson CRV, Wheeler CD, Winearls CG, Ford I on behalf of the PIVOTAL Study investigators 2018. Randomised controlled trial comparing high-dose versus low-dose intravenous iron supplementation in haemodialysis (PIVOTAL): study design and baseline data. Am J Nephrol. 48:260–8. 10.1159/000493551 PMC6262676 30304714

[14] Macdougall IC, White C, Anker SD, Bhandari S, Farrington K, Kalra PA, McMurray JJ, Murray H, Steenkamp R, Tomson CRV, Wheeler CD, Winearls CG, Ford I on behalf of the PIVOTAL Study investigators 2019. Randomised controlled trial comparing high-dose versus low-dose intravenous iron supplementation in hemodialysis (PIVOTAL). New Eng J Med. 380:447–58.

[15] Iain C, Macdougall IC, Aarau K, Block AH, Carrera F, Eckardt K-U, Gaillard C, Van Wyck D, Roubert B, Nolen JG, Roger SD, on behalf of the FIND-CKD Study Investigators (2014). FIND-CKD: a randomised trial of intravenous ferric carboxymaltose versus oral iron in patients with chronic kidney disease and iron deficiency anaemia. Nephrol Dial Transplant.

[16] Kalra PA, Bhandari S, Saxena S, Agarwal D, Wirtz G, Kletzmayr J, Thomsen LL, Coyne DW (2016). A randomised trial of iron isomaltoside 1000 versus oral iron in non-dialysis-dependent chronic kidney disease patients with anaemia. Nephrol Dial Transplant.

[17] Bhandari S, Allgar V, Lamplugh A, Macdougall KPI (2020). Protocol and baseline data of a multicentre prospective double blinded randomised study of intravenous iron on functional status in patients with chronic kidney disease. Am J Nephrol.

[18] Dukka H, Kalra PA, Wilkie M, Bhandari S, Davies SJ, Barrat J, Odudu A, Selby NM, McIntyre C, Robertson F, Taal MW (2019). Peritoneal ultrafiltration for heart failure; lessons learned from a randomised controlled trial. Perit Dial Int.

[19] Macdougall IC, Bircher IC, Eckardt KU, Obrador GT, Pollock CA, Stenvinkel P, Swinkels DW, Wanner C, Weiss G, Chertow GM, for Conference Participants (2016). Iron management in chronic kidney disease: conclusions from a “kidney disease: improving global outcomes” (KDIGO) controversies conference. Kidney Int.

[20] Mikhail A, Brown C, Williams JA, Mathrani V, Shrivastava R, Evans J, Isaac H, Bhandari S (2017). Renal association clinical practice guideline on Anaemia of Chronic Kidney Disease. BMC Nephrol.

[21] National Institute for Health and Clinical Excellence (2015). Chronic kidney disease: managing anaemia.

[22] Nuhu F, Bhandari S (2018). Oxidative stress and cardiovascular complications in CKD, the impact of anaemia. Iron Ther Targets Hum Diseases Pharm.

[23] Bhandari S, Pereira DIA, Chappell HF, Drakesmith H (2018). Intravenous irons: from basic science to clinical practice. Pharmaceuticals.

[24] Reddy V, Bhandari S, Seymour AM (2007). Myocardial function, energy provision and carnitine deficiency in experimental uraemia. J Am Soc Nephrol.

[25] Aksentijević D, Bhandari S, Seymour AM (2009). Insulin resistance and altered glucose transporter 4 expression in experimental uraemia. Kidney Int.

[26] Smith K, Semple D, Aksentijević D, Bhandari S, Seymour AM (2010). Functional and metabolic adaptation in uraemic cardiomyopathy. Front Biosci (Elite Ed).

[27] Toblli JE, Lombraña A, Duarte P, Di Genarro F (2007). Intravenous iron reduces NT pro brain natriuretic peptide in anaemia patients with chronic heart failure and renal insufficiency. J Am Coll Cardiol.

[28] Kalra PA, Bhandari S, Spyridon M, Davinson R, McCafferty K, Mikhail A, Reaich D, Lawman S, Moore J (2018). The NIMO UK study: is 1000 mg of intravenous iron enough to achieve Hb targets in pre-dialysis anaemia patients?. Nephrol Dial Transplant.

