# Supplementary material for: A multicentre prospective double blinded randomised controlled trial of intravenous iron (ferric Derisomaltose (FDI)) in Iron deficient but not anaemic patients with chronic kidney disease on functional status
Source: BMC Nephrol. 2021 Mar 30;22:115. doi: 10.1186/s12882-021-02308-y (PMC8010943; doi:10.1186/s12882-021-02308-y)
Supplement: Supplementary file 1 — Additional file 1: Supplementary Table 1. Summary of change in six-minute walk test (^MWT) baseline to 1 month and 3 months. Supplementary Table 2a. Summary of Minnesota Living with Heart Failure (MHLF) Questionnaire at baseline to 1 month and 3 months. Supplementary Table 2b. Summary of Restless legs score (RLS) Questionnaire at baseline to 1 month and 3 months. Supplementary Table 3a and b. Descriptive summary of Kidney Disease Quality of Life – Short form − 13 (KDQoL-SF) mean z transformed and normalised scores Questionnaire at baseline to 1 month and 3 months for total component summary measure (Physical Health and Mental Health individually and the total overall score). Initial analysis using ANCOVA and subsequent repeated measures analysis. Supplementary Table 4. Summary of Renal Function as assessed by serum creatinine (micromole/L). eGFR (ml/min/1.73m2), Cystatin C (g/L) and urinary proteinuria (mg/mmol) were similar in both groups and there was no significant change from baseline to 1 or 3 months. uACR – urinary abbumin;creatinine ratio; uPCR = urinary protein creatinine ratio. Supplementary Table 5. Summary of mean blood pressure (BP) in mmHG and Pulse wave velocity (PWV) measures including augmentation index (AiX) at baseline, 1 month and 3 months. Supplementary Table 6. Summary of Cardiac Biomarker; N terminal pro Brain natriuretic peptide (NT Pro BNP). [file 12882_2021_2308_MOESM1_ESM.docx]

**CONTENTS**

**Supplementary Tables**

**Supplementary Table 1**. Summary of change in six-minute walk test (^MWT) baseline to 1 month and 3 months

**Supplementary Table 2a**. Summary of Minnesota Living with Heart Failure (MHLF) Questionnaire at baseline to 1 month and 3 months

**Supplementary Table 2b**. Summary of Restless legs score (RLS) Questionnaire at baseline to 1 month and 3 months

**Supplementary Table 3a and b**. Descriptive summary of Kidney Disease Quality of Life – Short form -13 (KDQoL-SF) mean z transformed and normalised scores Questionnaire at baseline to 1 month and 3 months for total component summary measure (Physical Health and Mental Health individually and the total overall score). Initial analysis using ANCOVA and subsequent repeated measures analysis.

**Supplementary Table 4**. Summary of Renal Function as assessed by serum creatinine (micromole/L). eGFR (ml/min/1.73m^2^), Cystatin C (g/L) and urinary proteinuria (mg/mmol) were similar in both groups and there was no significant change from baseline to 1 or 3 months. uACR – urinary abbumin;creatinine ratio; uPCR = urinary protein creatinine ratio.

**Supplementary Table 5**: Summary of mean blood pressure (BP) in mmHG and Pulse wave velocity (PWV) measures including augmentation index (AiX) at baseline, 1 month and 3 months

**Supplementary Table 6**; Summary of Cardiac Biomarker; N terminal pro Brain natriuretic peptide (NT Pro BNP).

**Supplementary Table 1**. Summary of change in six-minute walk test baseline to 1 month and 3 months

|  | Group | n | Mean (SD) | Median  (IQR) | p-value |
| --- | --- | --- | --- | --- | --- |
| Change from baseline to 1 month | FDI | 22 | 3.5 (108.1) | 14.5 (-6, 38) | 0.952 |
|  | Placebo | 25 | 5.0 (58.8) | 1 (-29, 33) |  |
| Change from baseline to 3 months | FDI | 20 | 6.0 (89.1) | 29 (-36, 55) | 0.895 |
|  | Placebo | 24 | 1.9 (111.2) | 10.5 (-36, 45.5) |  |

**Supplementary Table 2a**. Summary of Minnesota Living with Heart Failure (MHLF) Questionnaire at baseline to 1 month and 3 months

|  |  | FDI | | | Placebo | | |  |
| --- | --- | --- | --- | --- | --- | --- | --- | --- |
|  |  | Mean | SD | n | Mean | SD | n | P value |
| MLHF | Baseline | 22.7 | 25.7 | 18 | 25.0 | 27.3 | 24 |  |
|  | 1 month | 20.6 | 24.7 | 22 | 20.9 | 23.9 | 23 | 0.569 |
|  | 3 months | 18.3 | 21.9 | 20 | 19.0 | 22.2 | 21 | 0.630 |

**Supplementary Table 2b**. Summary of Restless legs score (RLS) Questionnaire at baseline to 1 month and 3 months

|  | Group 1 | | | | |
| --- | --- | --- | --- | --- | --- |
|  | Ferric Derisomaltose | | Placebo | |  |
|  | Mean, n | SD | Mean, n | SD |  |
| RLS Baseline | 9.5 | 10.4 | 9.8 | 10.4 |  |
| RLS 1 month | 8.5 | 11.4 | 9.33 | 11 | p=NS |
| RLS 3 month | 7.5 | 9.5 | 6.9 | 9.9 | p=NS |

**Supplementary Table 3a and b**. Descriptive summary of Kidney Disease Quality of Life – Short form -13 (KDQoL-SF) mean z transformed and normalised scores Questionnaire at baseline to 1 month and 3 months for total component summary measure (Physical Health and Mental Health individually and the total overall score). Analysis for table 3a using ANCOVA and subsequent Table 3b repeated measures analysis.

|  | Group | | | | | | p-value |
| --- | --- | --- | --- | --- | --- | --- | --- |
|  | Placebo | | | Ferric Derisomaltose (FDI) | | |  |
|  | Mean | SD | n | Mean | SD | n |  |
| **Vitality** |  |  |  |  |  |  |  |
| Baseline | 39.7 | 10.7 | 27 | 43.3 | 9.9 | 20 |  |
| 1 month | 42.5 | 9.3 | 25 | 44.0 | 9.9 | 22 | 0.880 |
| 3 months | 41.4 | 9.9 | 24 | 44.1 | 10.0 | 21 | 0.475 |
| **Physical Health** |  |  |  |  |  |  |  |
| Baseline | 38.5 | 9.7 | 27 | 39.0 | 9.6 | 21 |  |
| 1 month | 39.6 | 9.5 | 25 | 40.9 | 9.0 | 22 | 0.659 |
| 3 months | 36.8 | 11.9 | 25 | 39.1 | 9.4 | 20 | 0.823 |
| **Mental** **Health** |  |  |  |  |  |  |  |
| Baseline | 42.2 | 10.2 | 27 | 42.3 | 11.2 | 22 |  |
| 1 month | 44.1 | 9.5 | 25 | 45.4 | 9.3 | 22 | 0.990 |
| 3 months | 42.8 | 10.3 | 24 | 44.6 | 10.6 | 21 | 0.936 |
| **Total score** |  |  |  |  |  |  |  |
| Baseline | 40.3 | 9.5 | 27 | 40.2 | 10.4 | 22 |  |
| 1 month | 41.8 | 9.2 | 25 | 43.2 | 8.5 | 22 | 0.795 |
| 3 months | 39.2 | 11.3 | 25 | 41.4 | 9.7 | 21 | 0.892 |

| Group |  | Mean | SE | 95% Confidence Interval | | p-value |
| --- | --- | --- | --- | --- | --- | --- |
|  |  |  |  | Lower | Upper |  |
| Vitality |  |  |  |  |  |  |
| FDI  n=13 | Baseline | 40.8 | 2.1 | 36.5 | 45.1 | 0.910 |
|  | 1 month | 43.2 | 2.0 | 39.1 | 47.3 |  |
|  | 3 months | 42.2 | 2.1 | 38.1 | 46.4 |  |
| Placebo  n=22 | Baseline | 41.7 | 2.7 | 36.1 | 47.3 |  |
|  | 1 month | 40.9 | 2.6 | 35.5 | 46.2 |  |
|  | 3 months | 44.4 | 2.7 | 39.0 | 49.9 |  |
| Physical Health |  |  |  |  |  |  |
| FDI  n=14 | Baseline | 39.3 | 1.9 | 35.5 | 43.2 | 0.499 |
|  | 1 month | 39.4 | 1.9 | 35.5 | 43.3 |  |
|  | 3 months | 37.9 | 2.3 | 33.2 | 42.5 |  |
| Placebo  n=23 | Baseline | 36.8 | 2.4 | 31.9 | 41.8 |  |
|  | 1 month | 37.9 | 2.5 | 32.9 | 42.9 |  |
|  | 3 months | 36.8 | 2.9 | 30.8 | 42.8 |  |
| Mental Health |  |  |  |  |  |  |
| FDI  n=14 | Baseline | 43.2 | 2.1 | 38.8 | 47.5 | 0.711 |
|  | 1 month | 44.4 | 2.1 | 40.1 | 48.7 |  |
|  | 3 months | 43.7 | 2.1 | 39.4 | 48.0 |  |
| Placebo  n=22 | Baseline | 42.1 | 2.7 | 36.7 | 47.6 |  |
|  | 1 month | 42.5 | 2.6 | 37.2 | 47.9 |  |
|  | 3 months | 43.8 | 2.6 | 38.4 | 49.2 |  |
| Total score |  |  |  |  |  |  |
| FDI  n=14 | Baseline | 41.0 | 1.9 | 37.1 | 44.8 | 0.703 |
|  | 1 month | 41.6 | 1.9 | 37.8 | 45.5 |  |
|  | 3 months | 40.1 | 2.2 | 35.8 | 44.5 |  |
| Placebo  n=23 | Baseline | 39.5 | 2.4 | 34.5 | 44.4 |  |
|  | 1 month | 40.2 | 2.4 | 35.2 | 45.2 |  |
|  | 3 months | 40.3 | 2.8 | 34.7 | 45.9 |  |

**Supplementary Table 4**. Summary of Renal Function as assessed by serum creatinine (micromole/L). eGFR (ml/min/1.73m^2^), Cystatin C (g/L) and urinary proteinuria (mg/mmol) were similar in both groups and there was no significant change from baseline to 1 or 3 months. uACR – urinary abbumin;creatinine ratio; uPCR = urinary protein creatinine ratio.

|  | Group | | | | |
| --- | --- | --- | --- | --- | --- |
|  | Ferric Derisomaltose | | Placebo | |  |
|  | Mean, n | SD | Mean, n | SD | P value |
| Serum Creatinine Baseline | 167.0, 26 | 40.2 | 204.9, 28 | 67.3 |  |
| Serum Creatinine 1 month | 165.0, 23 | 39.1 | 210.3, 26 | 68.0 | 0.207 |
| Serum Creatinine 3 month | 171.2, 21 | 45.3 | 207.8, 24 | 72.3 | 0.545 |
|  |  |  |  |  |  |
| eGFR Baseline | 33.2, 26 | 9.3 | 29.1, 28 | 9.9 |  |
| eGFR 1 month | 33.5, 22 | 9.9 | 28.1, 25 | 8.6 | 0.085 |
| eGFR 3 month | 32.1, 21 | 9.5 | 28.2, 23 | 8.6 | 0.644 |
|  |  |  |  |  |  |
| Cystatin C Baseline | 2.1, 26 | 0.5 | 2.4, 26 | 0.6 |  |
| Cystatin C 1 month | 2.2, 24 | 0.5 | 2.4, 26 | 0.7 | 0.835 |
| Cystatin C 3 month | 2.2, 24 | 0.5 | 2.3, 25 | 0.6 | 0.267 |
|  |  |  |  |  |  |
| uACR Baseline | 26.9, 13 | 40 | 94.8, 13 | 181.4 |  |
| uACR 1 month | 34.9, 11 | 50.2 | 42.6, 8 | 61.6 | 0.439 |
| uACR 3 month | 49.5, 11 | 71.7 | 42.5, 12 | 44.7 | 0.232 |
|  |  |  |  |  |  |
| uPCR Baseline | 51.9, 19 | 59.3 | 112.7, 21 | 164.8 |  |
| uPCR 1 month | 44.3, 16 | 80.9 | 59.9, 20 | 79.8 | 0.636 |
| uPCR 3 months | 58.1, 16 | 92.3 | 70.0, 17 | 102.1 | 0.725 |

**Supplementary Table 5**: Summary of mean blood pressure (BP) in mmHG and Pulse wave velocity (PWV) measures including augmentation index (AiX) at baseline, 1 month and 3 months

|  | Group | | | | |
| --- | --- | --- | --- | --- | --- |
|  | Ferric Derisomaltose | | Placebo | |  |
|  | Mean, n | SD | Mean, n | SD | P value |
| PWV measurement Baseline | 8.3, 26 | 2.8 | 8.3, 28 | 3.6 |  |
| PWV measurement 1 month | 8.0, 22 | 2.0 | 8.3, 26 | 2.0 | 0.371, 0.638 |
| PWV measurement 3 month | 8.3, 22 | 1.9 | 9.7, 24 | 6.6 | 0.184, 0.287 |
|  |  |  |  |  |  |
| AiX measurement Baseline | 25.4, 26 | 10.7 | 24.1, 28 | 10.8 |  |
| AiX measurement 1 month | 21.0, 22 | 10.5 | 21.6, 26 | 12.4 | 0.638, 0.814 |
| AiX measurement 3 month | 25.5, 22 | 10.6 | 24.3, 23 | 11.8 | 0.287, 0.716 |
|  |  |  |  |  |  |
| Systolic BP baseline | 138.2, 26 | 19.9 | 129.4, 28 | 18.4 |  |
| Systolic BP 1 month | 133.8, 23 | 22.3 | 130.5, 25 | 11.6 | 0.510 |
| Systolic BP 3 months | 134.2, 22 | 13.7 | 130.0, 25 | 16.2 | 0.875 |
|  |  |  |  |  |  |
| Diastolic BP baseline | 78.5, 26 | 10.7 | 76.2, 28 | 11.8 |  |
| Diastolic BP 1 month | 78.3, 23 | 12.0 | 73.6, 25 | 10.8 | 0.417 |
| Diastolic BP 3 months | 79.9, 22 | 9.0 | 73.6, 25 | 13.7 | 0.257 |

**Supplementary Table 6**; Summary of Cardiac Biomarker; N terminal pro Brain natriuretic peptide (NT Pro BNP) ng/L.

| NT pro BNP (ng/L) | Group | | | | |
| --- | --- | --- | --- | --- | --- |
|  | Ferric Derisomaltose | SD | Placebo | SD | P Value |
| Baseline | 422.5, 25 | 881.9 | 545.4, 26 | 1569.5 |  |
| 1 month | 242.5, 24 | 209.1 | 608.8, 27 | 1891.0 | 0.371 |
| 3 month | 278.5, 23 | 227.9 | 505.6, 27 | 1578.2 | 0.184 |
